# Supplementary figures and images for: A choline-releasing glycerophosphodiesterase essential for phosphatidylcholine biosynthesis and blood stage development in the malaria parasite
Source: eLife. 2022 Dec 28;11:e82207. doi: 10.7554/eLife.82207 (PMC9886279; doi:10.7554/eLife.82207)

Figure S3B – Source data

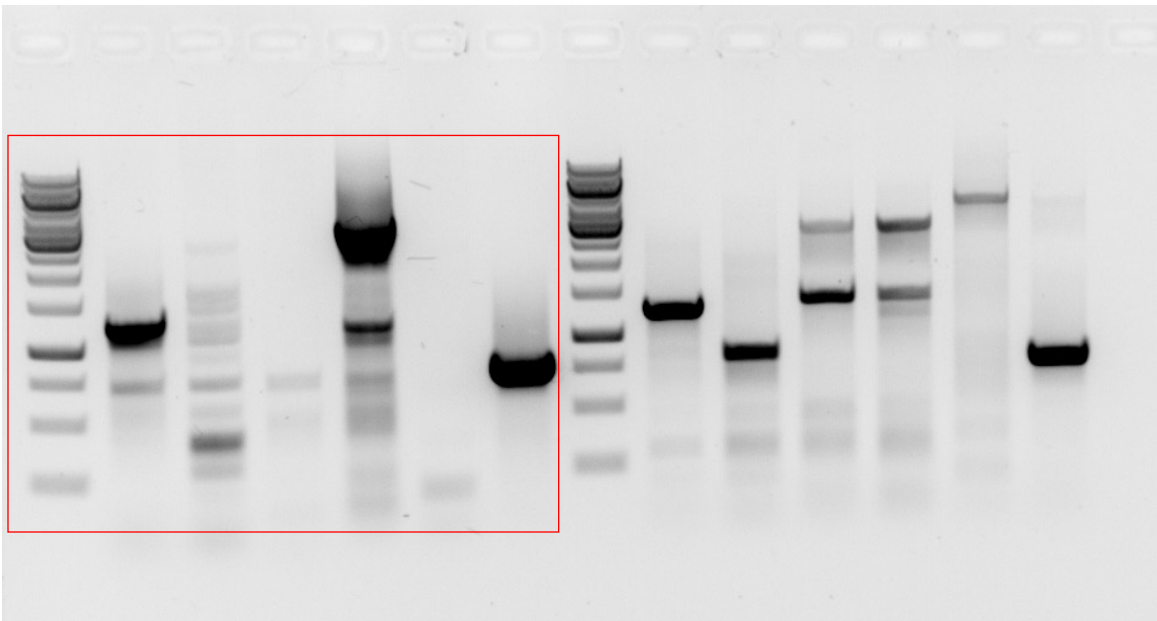

Figure S3C – Source data

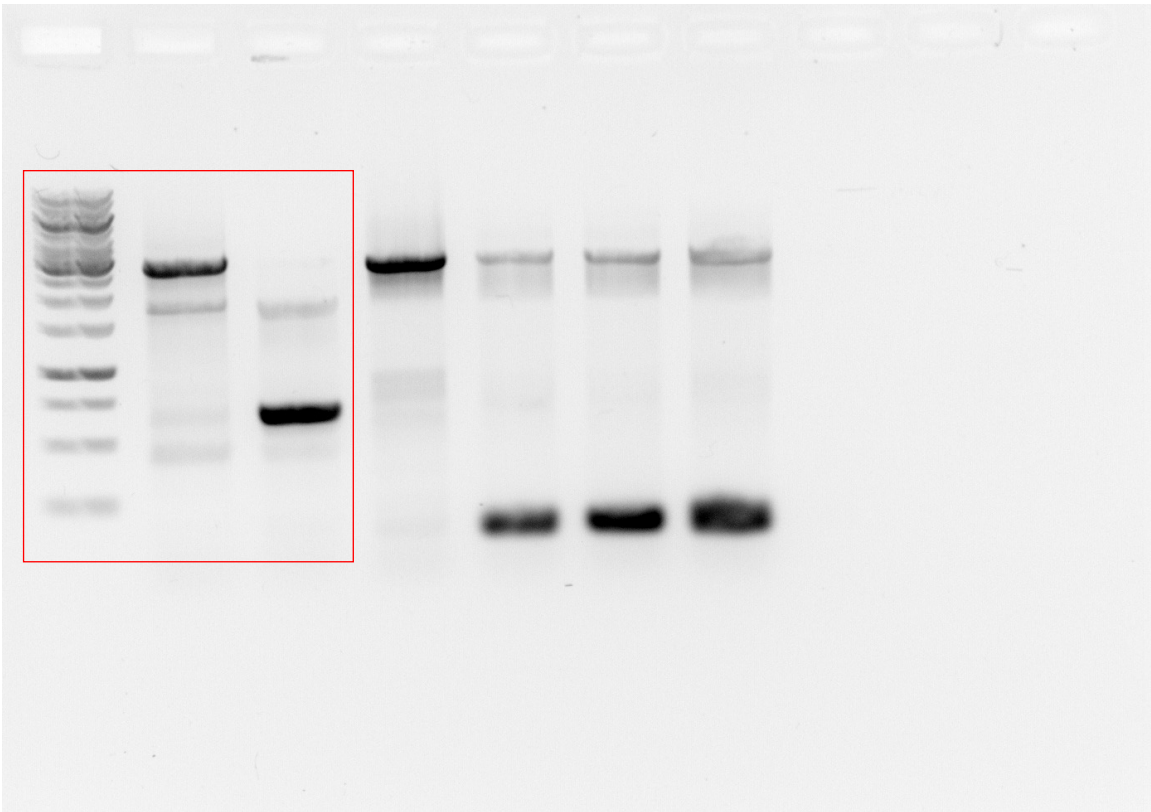

Figure S3D – Source data

anti-HA

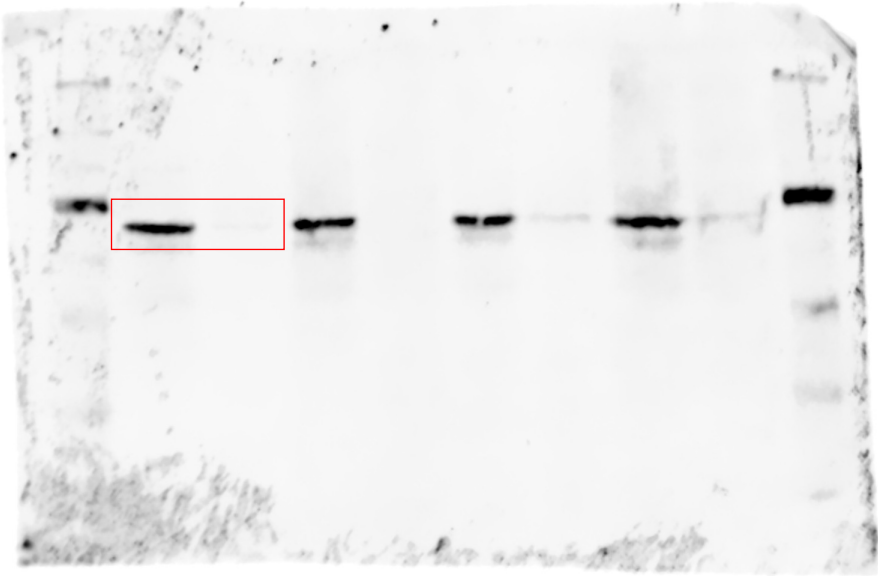

anti-Aldolase

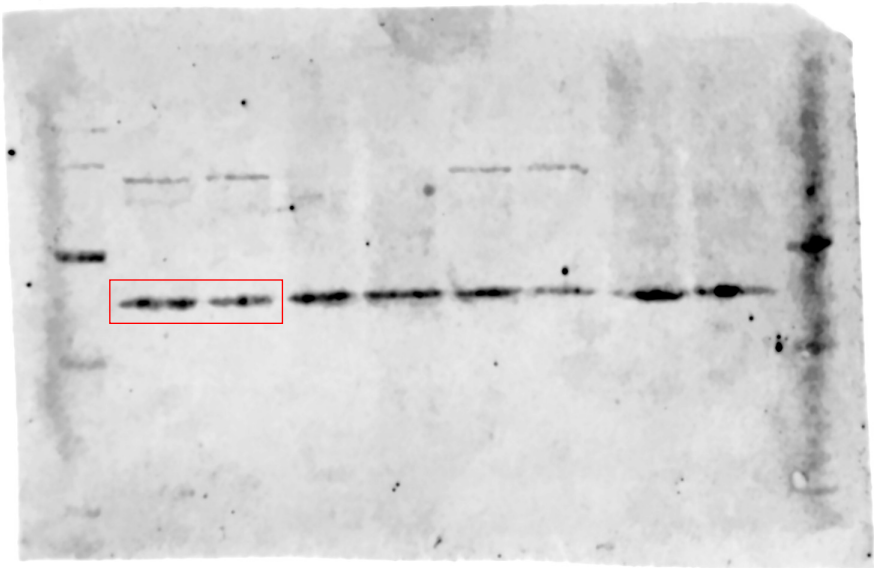

Supplement: Figure 2—source data 1. [file elife-82207-fig2-data1.zip › Figure2_sourcedata/Figure2_figsuppl3_orig_labelled.pdf]

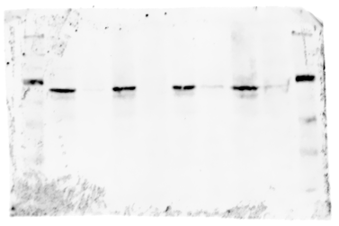

Supplement: Figure 2—source data 1. [file elife-82207-fig2-data1.zip › Figure2_sourcedata/Figure2_figsuppl3D_HA_original.png]

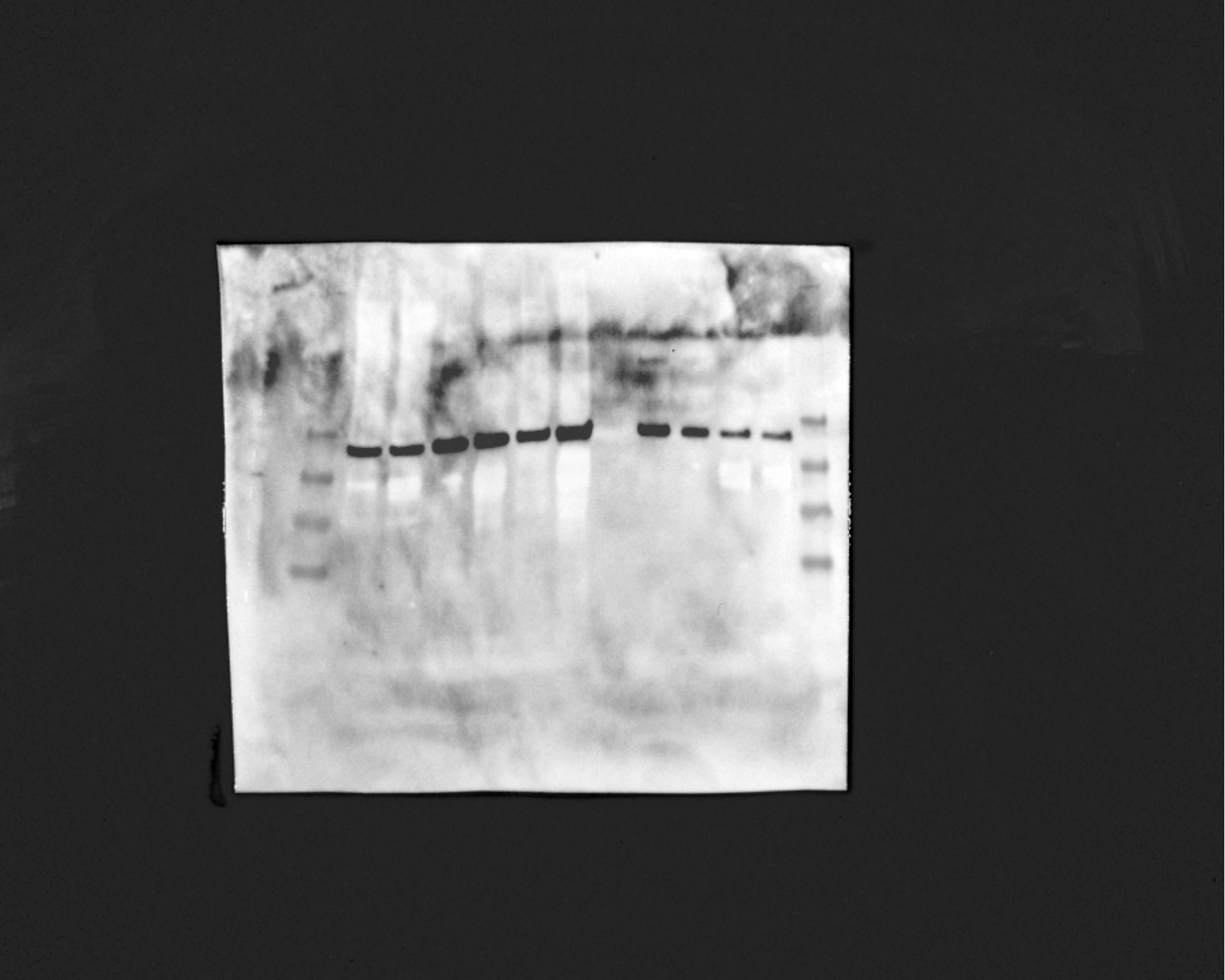

Supplement: Figure 2—source data 1. [file elife-82207-fig2-data1.zip › Figure2_sourcedata/Figure2D-HSP70original.tif]

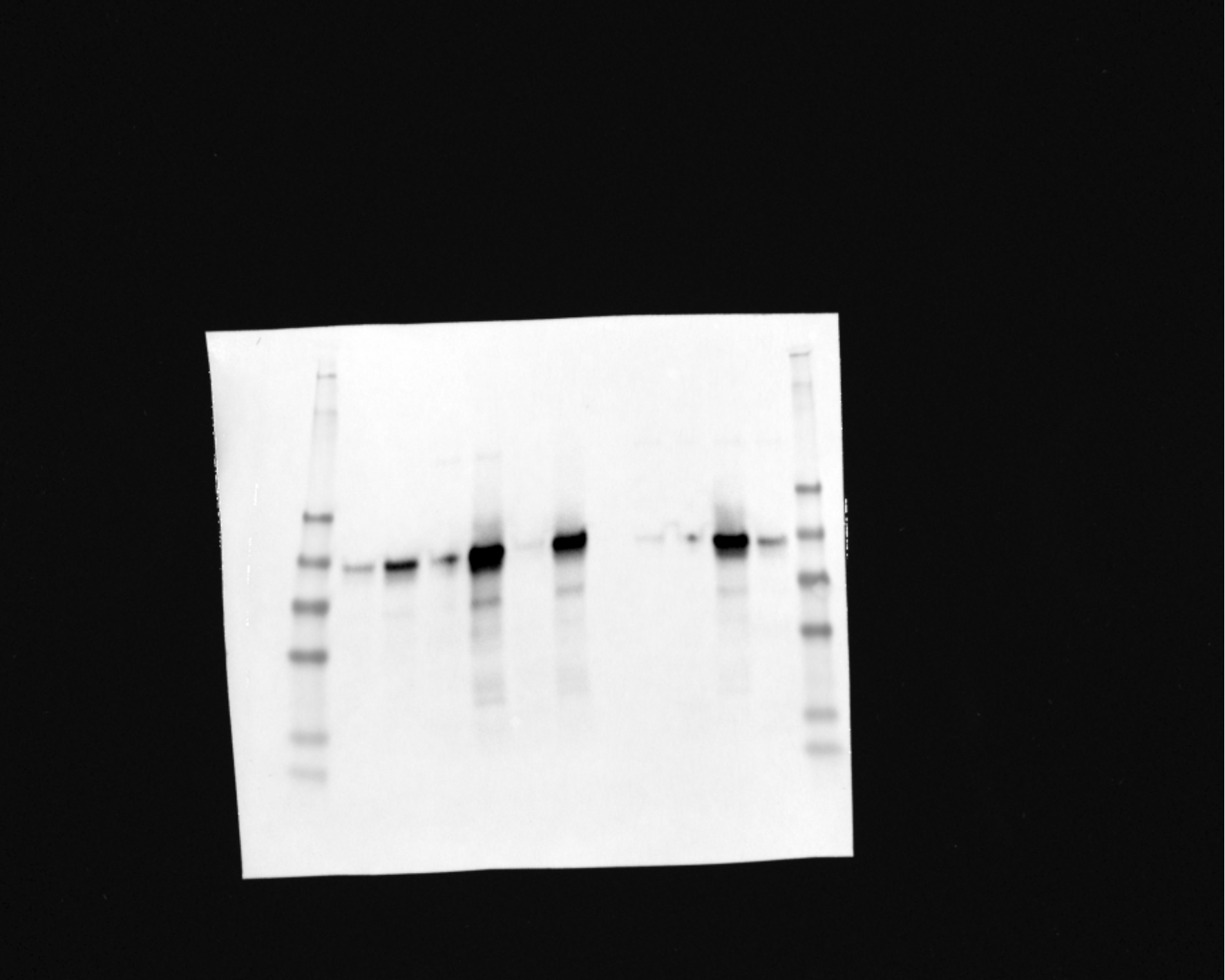

Supplement: Figure 2—source data 1. [file elife-82207-fig2-data1.zip › Figure2_sourcedata/Figure2D-HAoriginal.tif]

B4

B8

+

-

+

-

kbp

2.0 -

1.0 -

0.2 -

- Non-excised  
(1,719 bp)

- Excised  
(126 bp)

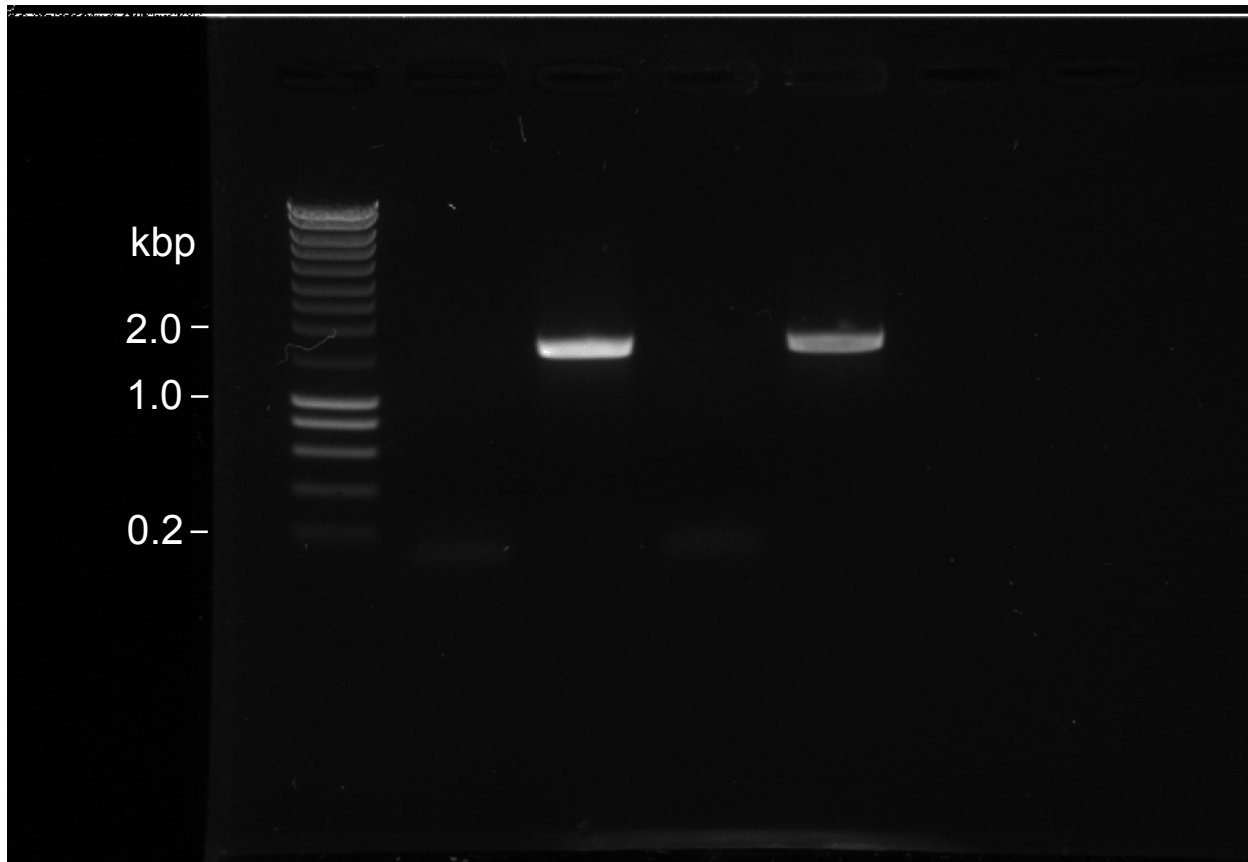

Supplement: Figure 2—source data 1. [file elife-82207-fig2-data1.zip › Figure2_sourcedata/Figure2C-original_labelled.pdf]

Figure S1B – Source data

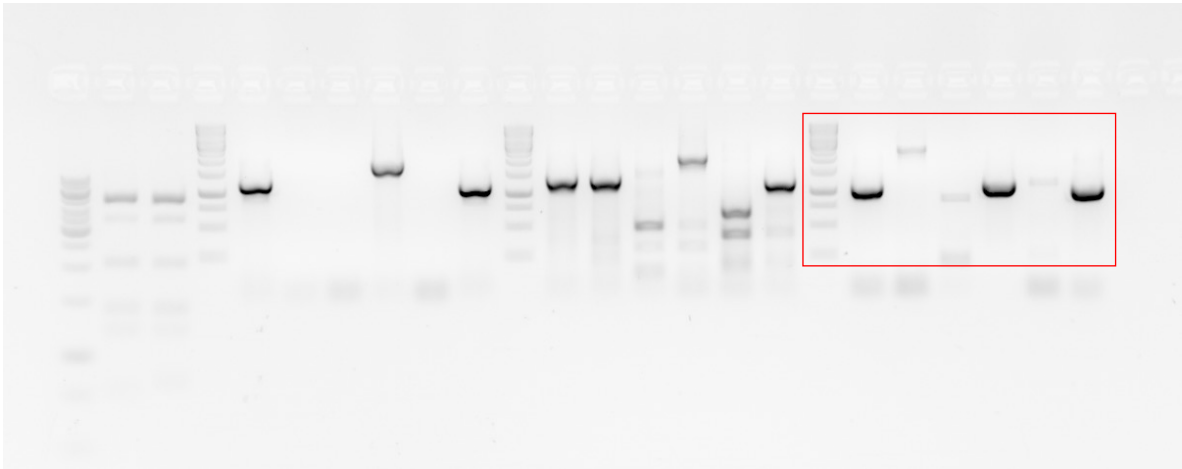

Supplement: Figure 2—source data 1. [file elife-82207-fig2-data1.zip › Figure2_sourcedata/Figure2-figsuppl1-orig_labelled.pdf]

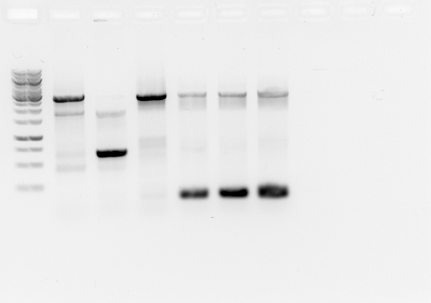

Supplement: Figure 2—source data 1. [file elife-82207-fig2-data1.zip › Figure2_sourcedata/Figure2_figsuppl3C_original.png]

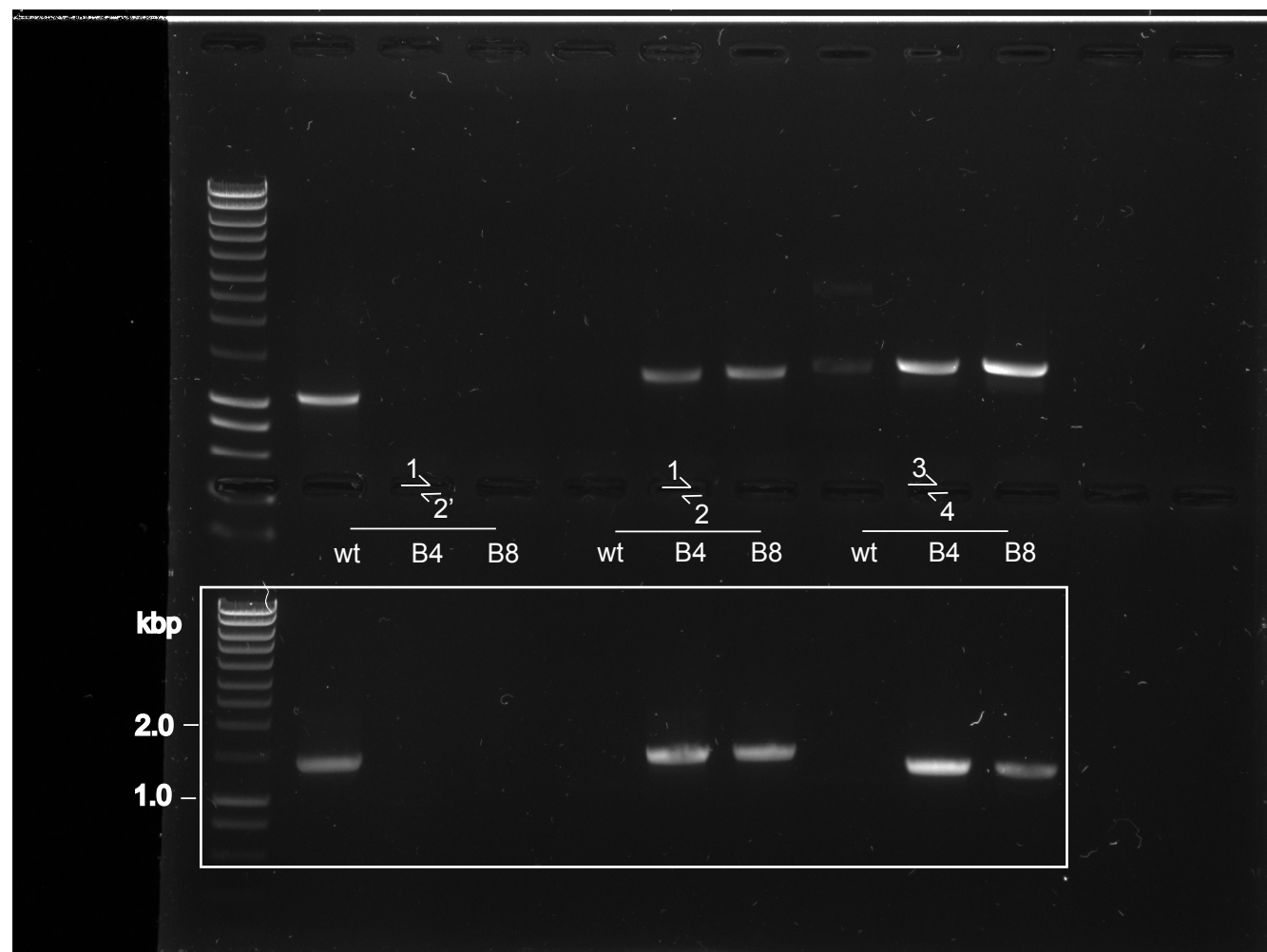

Supplement: Figure 2—source data 1. [file elife-82207-fig2-data1.zip › Figure2_sourcedata/Figure2_figuresuppl2_original_labelled.pdf]

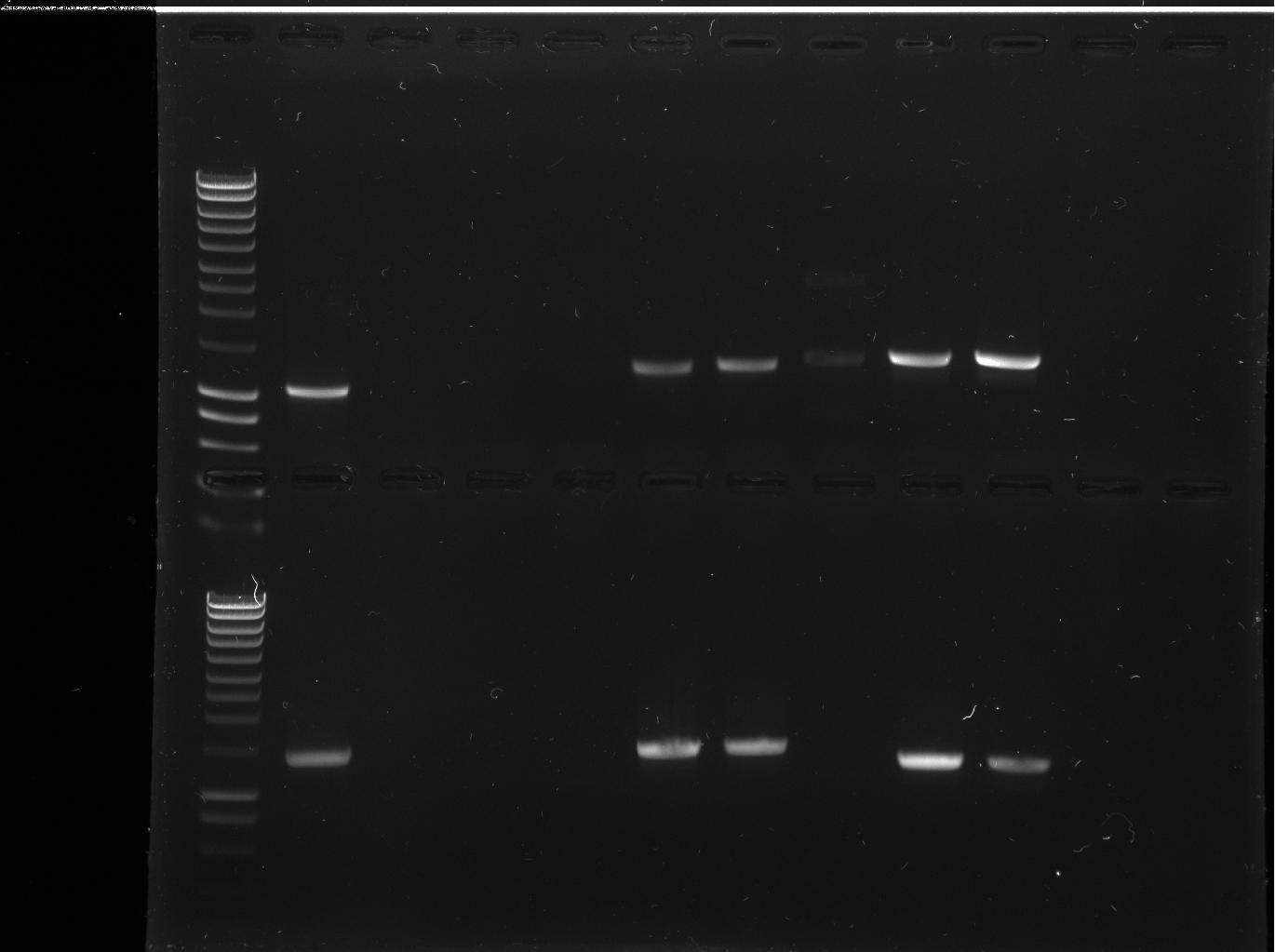

Supplement: Figure 2—source data 1. [file elife-82207-fig2-data1.zip › Figure2_sourcedata/Figure2_figuresuppl2_original.tif]

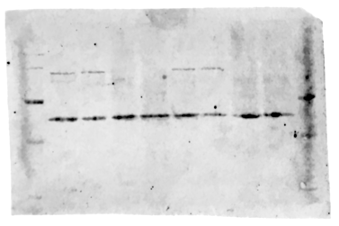

Supplement: Figure 2—source data 1. [file elife-82207-fig2-data1.zip › Figure2_sourcedata/Figure2_figsuppl3D_aldolase_original.png]

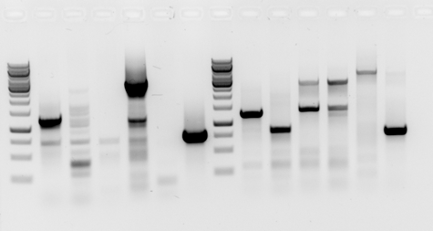

Supplement: Figure 2—source data 1. [file elife-82207-fig2-data1.zip › Figure2_sourcedata/Figure2_figsuppl3B_original.png]

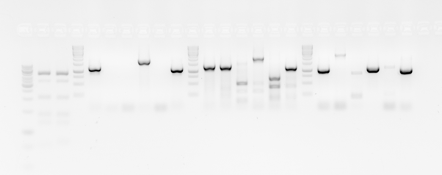

Supplement: Figure 2—source data 1. [file elife-82207-fig2-data1.zip › Figure2_sourcedata/Figure2-figuresuppl1_original.png]

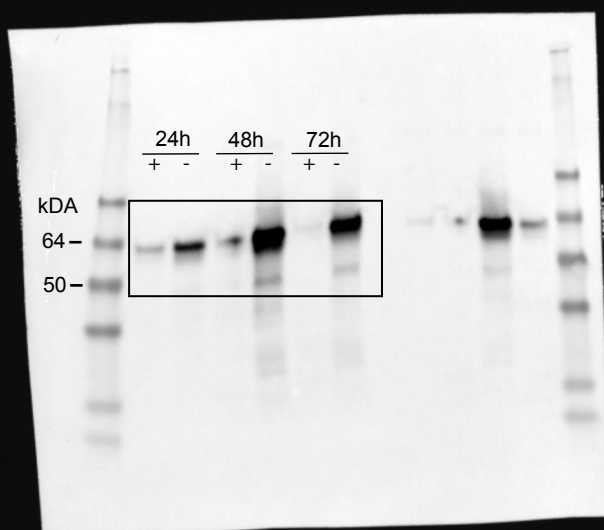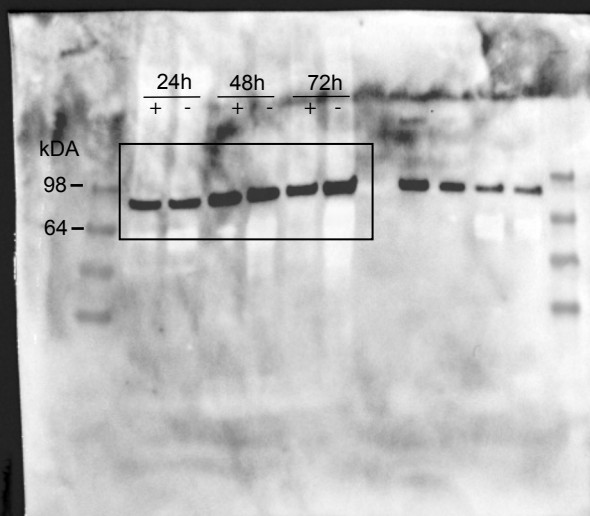

Supplement: Figure 2—source data 1. [file elife-82207-fig2-data1.zip › Figure2_sourcedata/Figure2D-original_labelled.pdf]

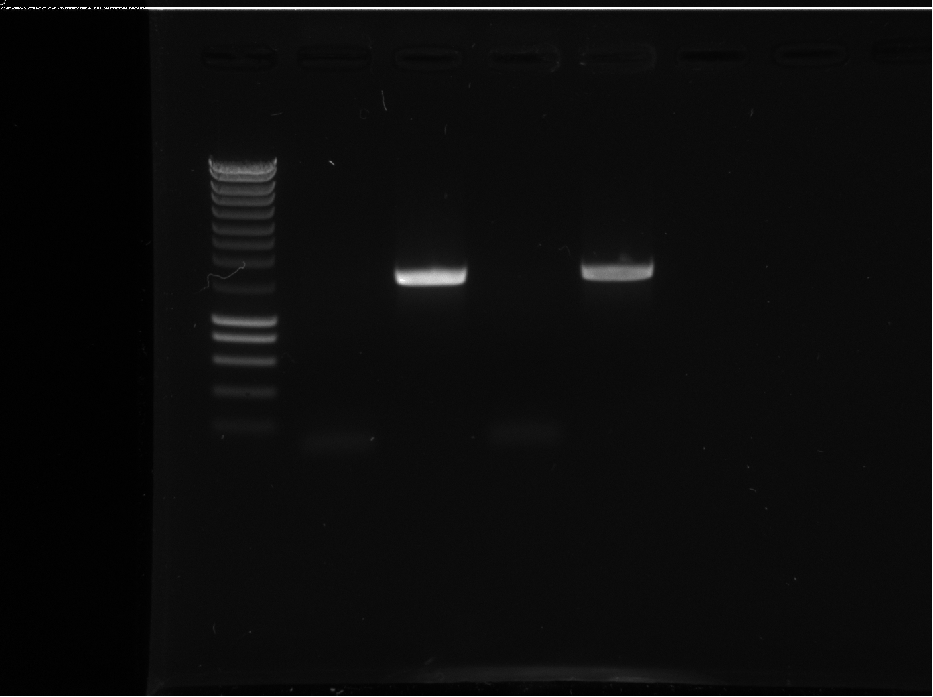

Supplement: Figure 2—source data 1. [file elife-82207-fig2-data1.zip › Figure2_sourcedata/Figure2C-original.tif]

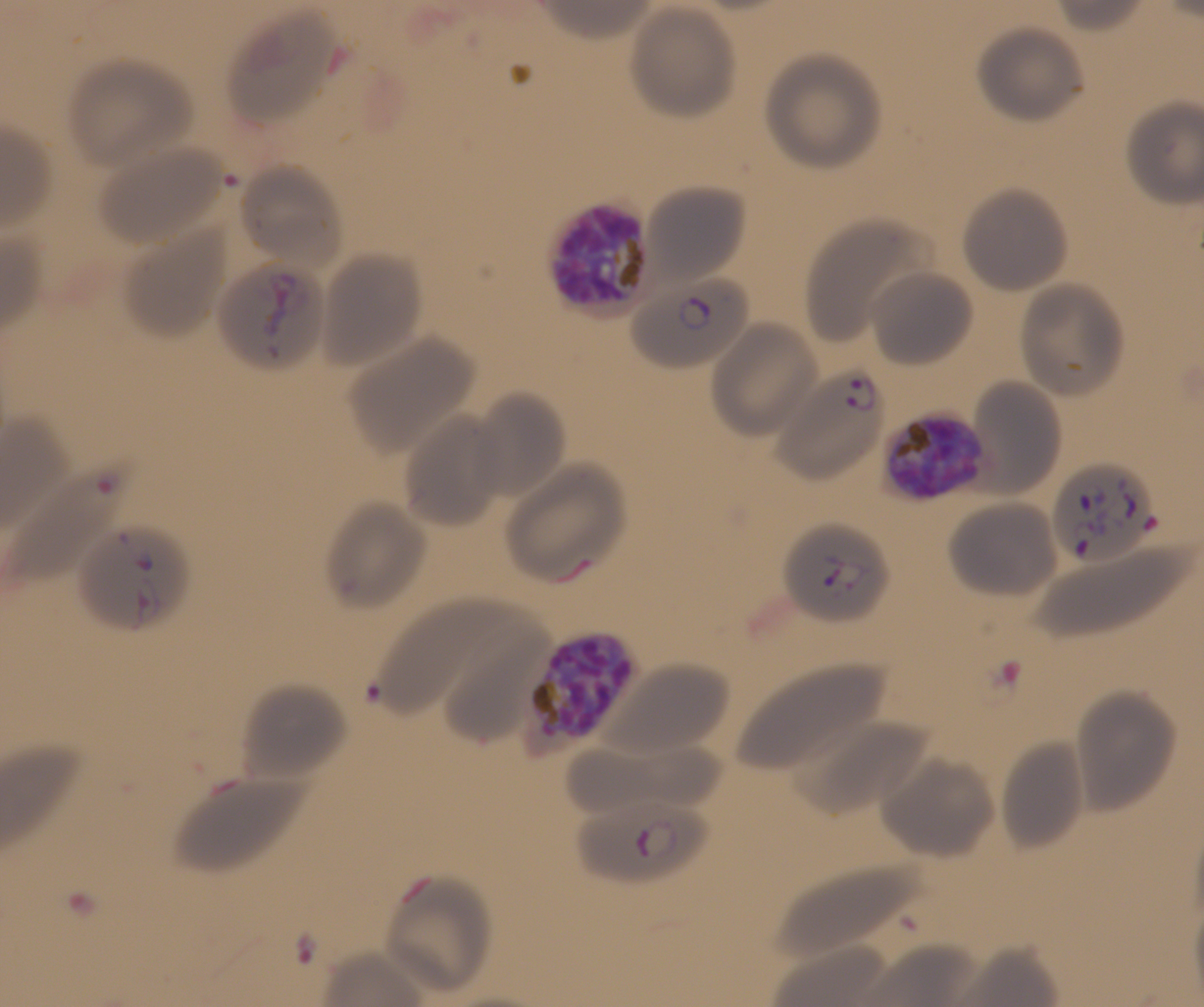

Supplement: Figure 4—source data 1. [file elife-82207-fig4-data1.zip › Figure4_sourcedata/Figure4E_G1+Cho48h-sourcedata.png]

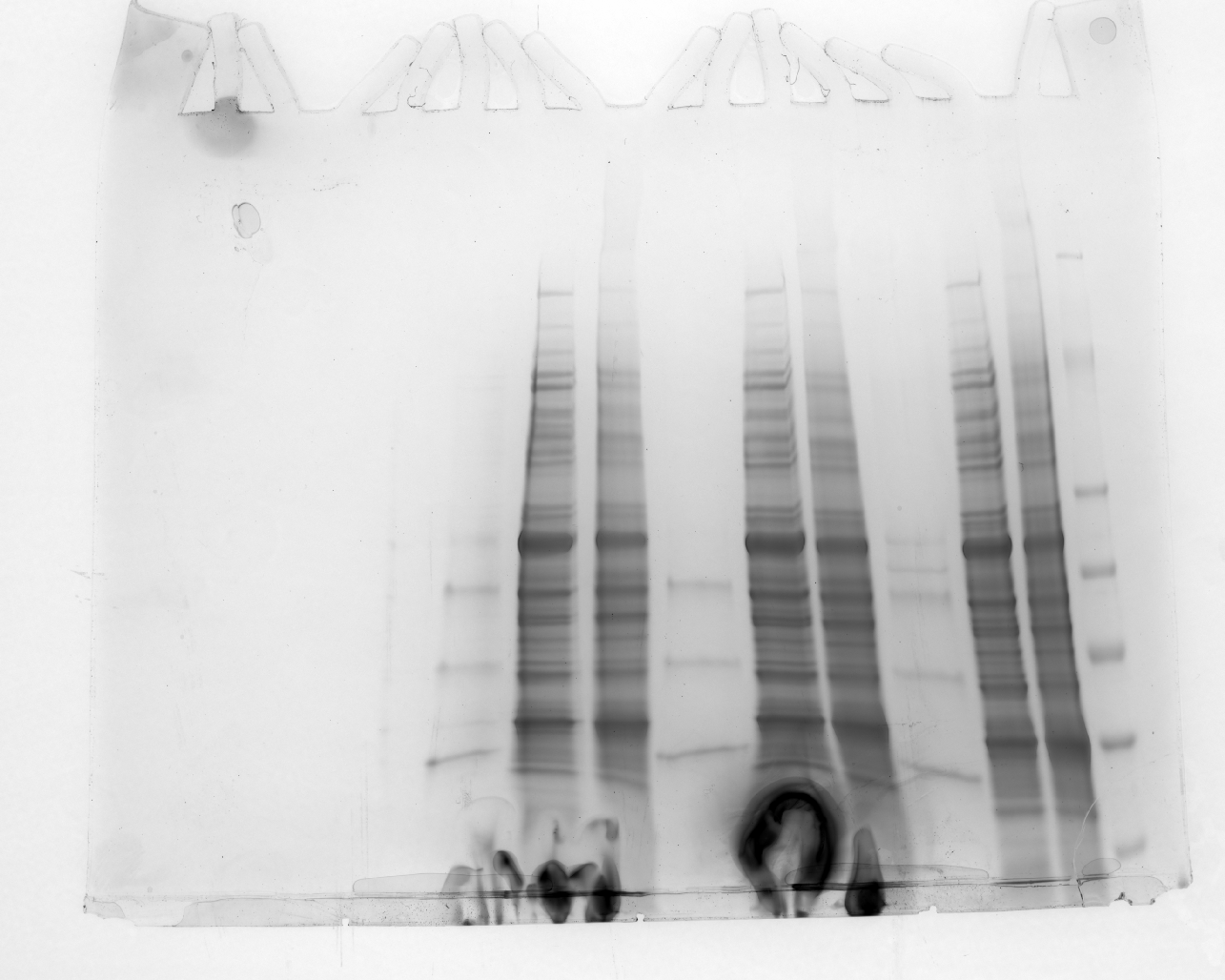

Supplement: Figure 6—source data 1. [file elife-82207-fig6-data1.zip › Figure6_sourcedata/Figure6_figuresuppl2A_original.tif]

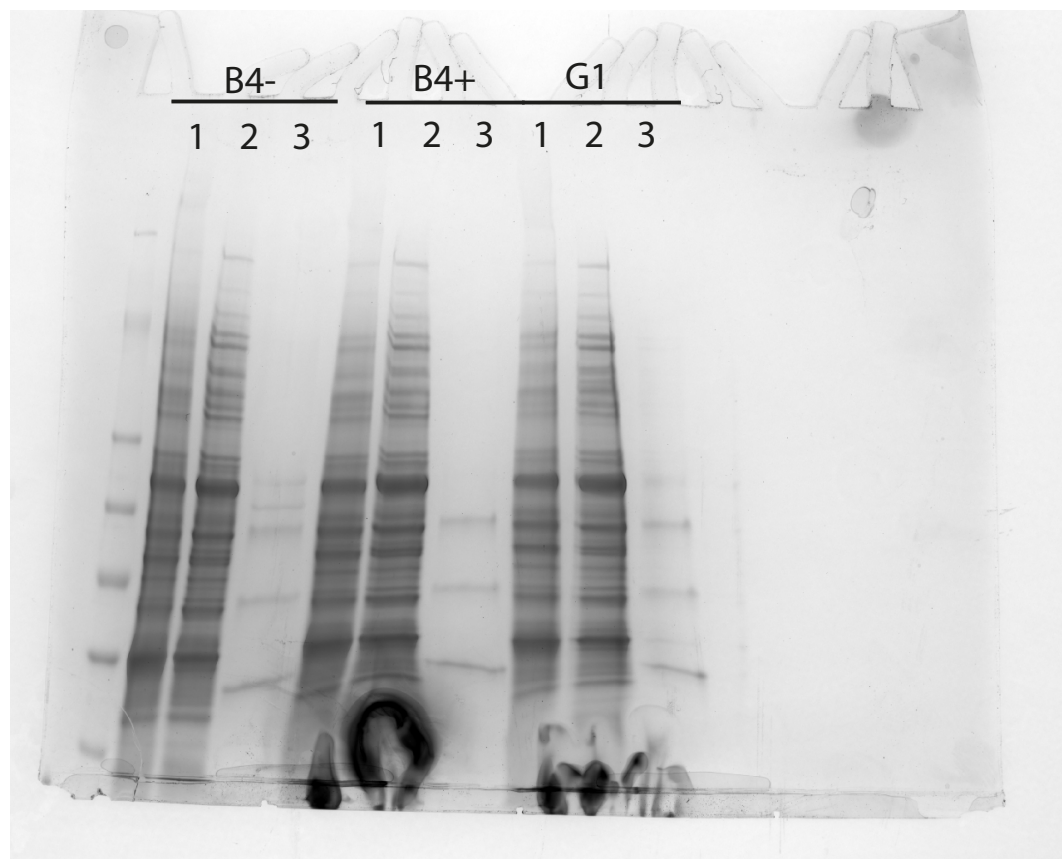

1. saponin lysate
2. supernatant
3. bound fraction

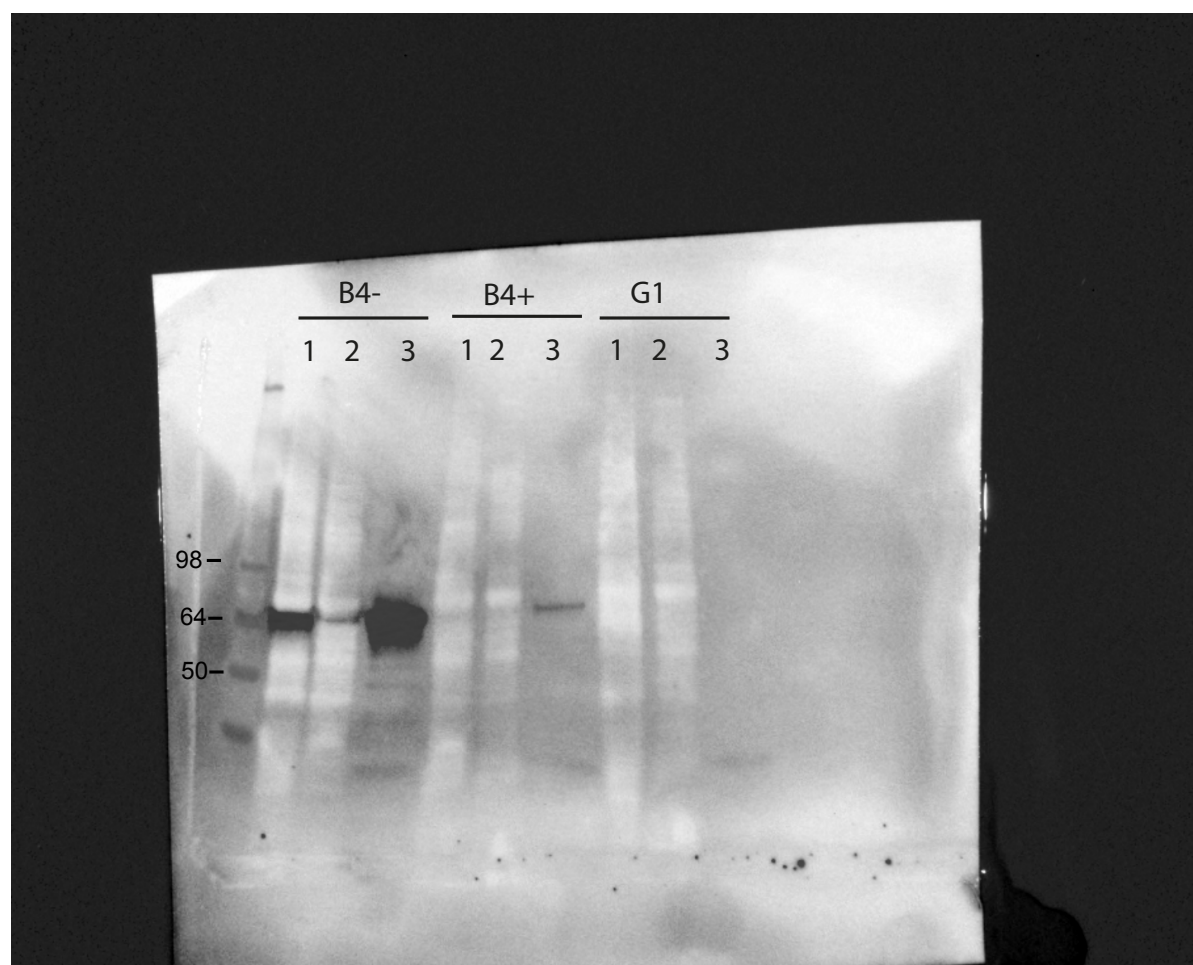

Supplement: Figure 6—source data 1. [file elife-82207-fig6-data1.zip › Figure6_sourcedata/Figure6_figuresuppl2_original_labelled.pdf]

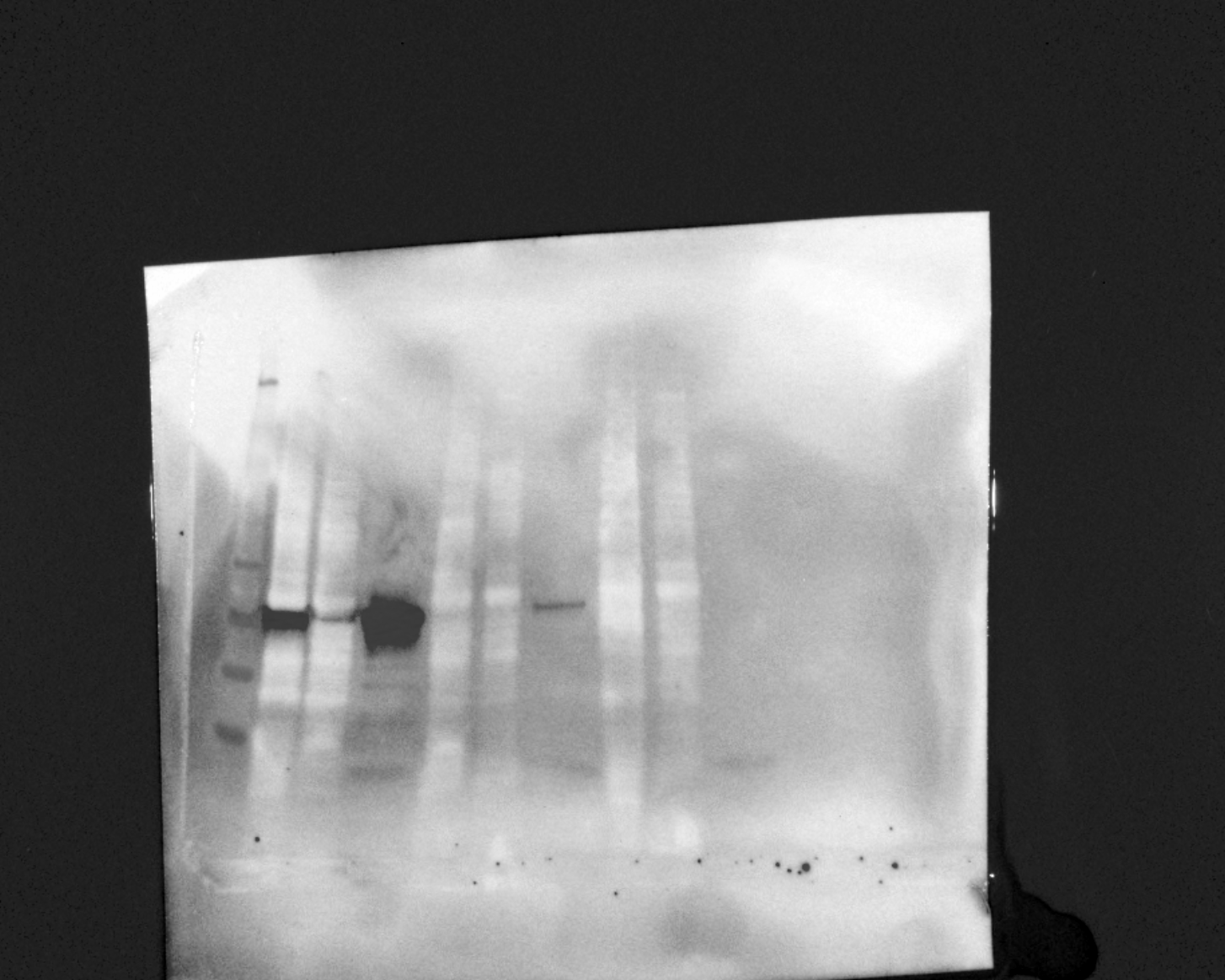

Supplement: Figure 6—source data 1. [file elife-82207-fig6-data1.zip › Figure6_sourcedata/Figure6_figuresuppl2B_original.tif]

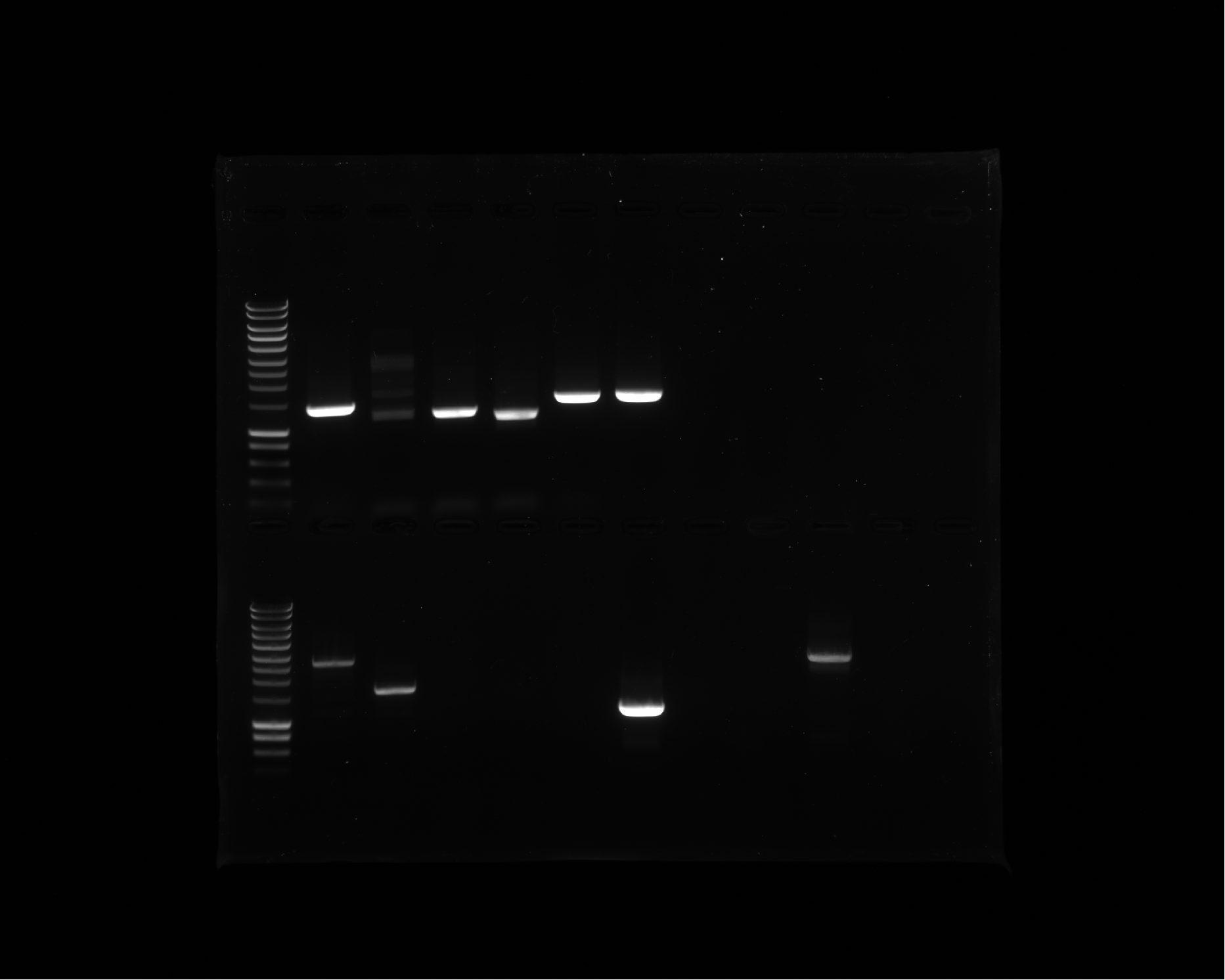

Supplement: Figure 7—source data 1. [file elife-82207-fig7-data1.zip › Figure7_sourcedata/Figure7_figuresuppl1_original_labelled.tif]

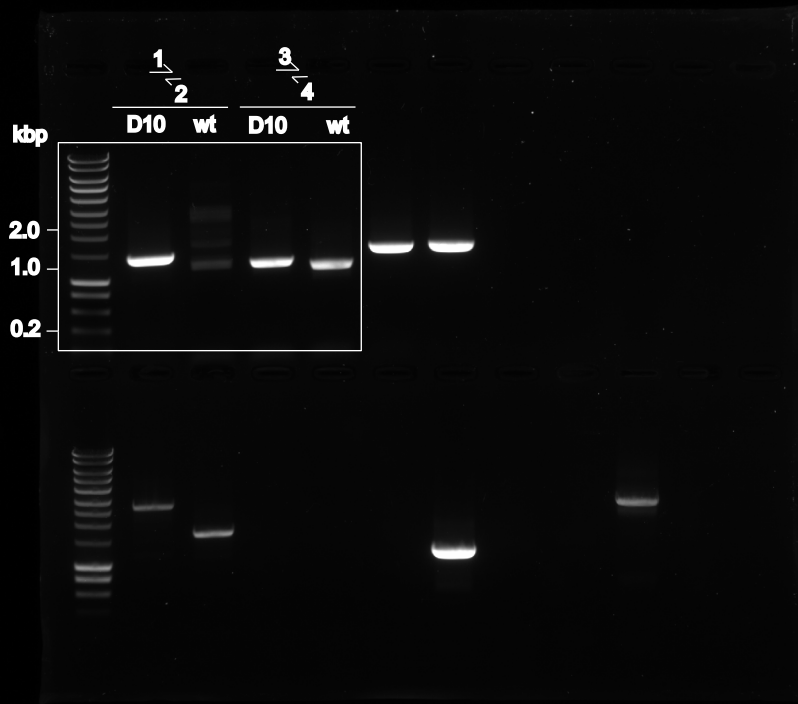

Supplement: Figure 7—source data 1. [file elife-82207-fig7-data1.zip › Figure7_sourcedata/Figure7_figuresuppl1_original_labelled.pdf]
